# A Rare Case of Bilateral Adrenal Hemorrhage

**DOI:** 10.7759/cureus.2830

**Published:** 2018-06-18

**Authors:** Zainab Fatima, Usman Tariq, Amina Khan, Muhammad Saad Sohail, Abu Baker Sheikh, Shimron I Bhatti, Kamran Munawar

**Affiliations:** 1 Medicine, Shifa International Hospital, Islamabad, PAK; 2 Research Assistant, Yale University School of Medicine, New Haven, USA; 3 Shifa Tameer E Millat University, Shifa International Hospital, Islamabad, PAK; 4 Internal Medicine, Shifa International Hospital, Islamabad, PAK; 5 Orthopaedics, Shifa International Hospital, Islamabad, PAK; 6 Internal Medicine, Shifa College of Medicine, Islamabad, PAK

**Keywords:** bilateral adrenal hemorrhage, adrenal hemorrhage, secondary to corticosteroids, rare

## Abstract

Bilateral adrenal hemorrhage (BAH) is a rare but potentially fatal entity that carries a mortality rate of 15%. Most cases are associated with sepsis, antiphospholipid syndrome, the use of anticoagulants, as well as trauma and surgery. In this case report, we present a case of BAH in a previously healthy man with a recent history of corticosteroid use. Our case emphasizes the ambiguous clinical presentation of BAH, which poses a challenge in the establishment of a correct diagnosis. We also illustrate the pathophysiology, diagnosis, and subsequent therapeutic approach to this rare clinical entity.

## Introduction

Adrenal hemorrhage is a rare condition associated with issues such as myocardial infarction, congestive heart failure, infection, trauma, surgery, use of anticoagulants and autoimmune etiologies such as antiphospholipid syndrome. However, cases with no discernible cause have also been reported in the literature. Patients with adrenal hemorrhage can present with non-specific symptoms, or overt manifestations of shock, adrenal insufficiency, and acute adrenal crisis [1].

We present the case of a middle-aged man with no known comorbidities, who presented to our clinical setting with a prior history of uveitis, which was being managed with oral corticosteroids for the last two weeks. He presented to our clinical setting with the complaints of persistent lethargy and proximal muscle weakness. Following an unsteady course in the inpatient setting (that was characterized by altered mentation and persistently low blood pressure), he was subjected to a computed tomography (CT) scan, which showed bilateral adrenal hemorrhages (BAH). An exhaustive inspection into any inciting cause was unrevealing. The non-specific presentation culminated in varying complexities and ultimately lead to the patient’s demise. This case illustrates the difficulty in making the diagnosis of BAH with atypical presentation. Patients with this disease require prompt initiation of treatment for better clinical outcomes.

## Case presentation

A 47-year-old male with no known comorbidities was started on a course of oral prednisone (60 mg once daily) following a routine diagnosis of bilateral anterior uveitis, with a consequential improvement in his visual acuity. In the ensuing two weeks, he developed generalized weakness and fatigue, which hindered his ability to walk without assistance. This prompted his visit to our emergency department. In a detailed interview, he did not complain of any chest pain, dyspnea, altered bowel habits, previous syncopal episodes, headaches, dizziness, or prior substance abuse. 

Initial assessment disclosed a patient with bilateral periorbital puffiness, who was visibly lethargic but alert and well-oriented, with a Glasgow Coma Scale (GCS) score of 15/15. His heart rate was 80 beats per minute with a blood pressure of 70/50 mm Hg, a respiratory rate of 20 per minute and a temperature of 98.6°F. An extensive neurological exam revealed 4/5 power in all muscle groups, intact perception to pain, touch, and proprioception, an absence of cerebellar signs or impaired plantar reflexes. An ophthalmological examination revealed normal visual acuity, pupillary reflexes, extraocular movements and fundoscopic examination.

In lieu of the severe hypotension, the patient was admitted to the medical floor and started on a combination of intravenous fluids, norepinephrine, hydrocortisone (100 mg every eight hours) as well as tazobactam/piperacillin (4.5 g every eight hours) to treat for any underlying sepsis. His initial laboratory investigations are shown in Table 1, and Table 2 shows the trend of laboratory investigations over a period of a next few days.

**Table 1:** Laboratory investigations. CPK: Creatine phosphokinase; CK-MB: Creatine kinase-muscle/brain; ALT: Alanine aminotransferase; CRP: C-reactive protein; ACTH: Adrenocorticotropic hormone; TSH; Thyroid stimulating hormone; T4: Thyroxine; T3: Triiodothyronine; C-ANCA: Cytoplasmic antineutrophil cytoplasmic antibodies; P-ANCA: Perinuclear anti-neutrophil cytoplasmic antibodies; HIV: Human immunodeficiency virus; ACE: Angiotensin converting enzyme; IgM: Immunoglobulin M.

Investigations	Value	Normal reference range
Serum sodium	137 mEq/L	136-145 mEq/L
Serum potassium	4.2 mEq/L	3.5-5.1 mEq/L
Serum chloride	108 mEq/L	98-107 mEq/L
Serum bicarbonate	15 mEq/L	22-29 mEq/L
Serum creatinine	0.72 mg/dL	0.72-1.25 mg/dL
Blood glucose random	290 mg/dL	<200 mg/dL
Hemoglobin	14.8 g/dL	13.5-18.0 g/dL
White blood cells	19500/µL	4000-10500/µL
Platelets	171000/µL	150,000-450,000/µL
Blood urea nitrogen	59 mg/dL	8.9-20.6 mg/dL
CK-MB	7.2 ng/mL	Up to 7.2 ng/mL
Troponin I	160 pg/mL	Up to 34.2 pg/mL
Creatine phosphokinase (CPK)	35 U/L	30-200 U/L
CK-MB (second set)	4.6 ng/mL	Upto 7.2 ng/mL
Troponin I (second set)	131.6 pg/mL	Up to 34.2 pg/mL
ALT	47 U/L	0-55 U/L
CRP	33.09 mg/L	Up to 5.0 mg/L
Serum albumin	1.97 g/dL	3.5-5.0 g/dL
Serum calcium	7.49 mg/dL	8.4 -10.2 mg/dL
Serum cortisol (evening)	59.7 ug/dL	2.9-17.3 ug/dL
ACTH	4.17 pg/mL	7.2-63.3 pg/mL
Serum TSH	0.16 uIU/mL	0.35-4.94 uIU/mL
Free T4	0.4 ng/dL	0.7-1.48 ng/dL
Free T3	1.29 pg/mL	2.0-4.4 pg/mL
Luteinizing hormone (LH)	2.86 mIU/mL	1.14-8.75 mIU/mL
Follicle stimulating hormone (FSH)	3.02 mIU/mL	0.95-11.95 mIU/mL
Prolactin	9.81 ng/mL	3.46-19.40 ng/mL
Serum procalcitonin	0.17 ng/mL	< 0.10 ng/mL
C-ANCA	<0.1 AU	Negative <5 AU
P-ANCA	<0.1 AU	Negative <5 AU
Anti-HIV antibody	Non-reactive	
ACE	97 U/L	Upto 52 U/L
D-Dimers	812 ng/mL	Upto 250 ng/mL
Serum lipase	85 U/L	8-78 U/L
Serum amylase	50 U/L	20-125 U/L
Anti-cardiolipin IgM	15.5 MPL U/mL	13.0-14.9 MPl U/mL= equivocal 15-39.9 MPI U/mL= low positive 40-79.9 MPI U/mL= moderate positive >80 = strongly positive

**Table 2:** Trend of laboratory investigations over the next few days. CRP: C-reactive protein

	Date				
Investigations	12/4/18	13/4/18	14/4/18	15/4/18	16/4/18
Serum sodium	137 mEq/L	139 mEq/L	138 mEq/L	139 mEq/L	138 mEq/L
Serum potassium	4.2 mEq/L	3.2 mEq/L	3.3 mEq/L	3.6 mEq/L	3.6 mEq/L
Serum bicarbonate	15 mEq/L	18 mEq/L	15 mEq/L	15 mEq/L	14 mEq/L
Serum creatinine	0.72 mg/dL	0.55 mg/dL	0.57 mg/dL	0.64 mg/dL	0.67 mg/dL
Blood urea nitrogen	59 mg/dL	29 mg/dL	25 mg/dL	26 mg/dL	32 mg/dL
CRP	33 mg/L	-	26.53 mg/L	16 mg/L	9.63 mg/L
White blood cell count	19500/µL	15300/µL	15300/µL	17500/µL	17400/µL
Hemoglobin	14.8 g/dL	12.0 g/dL	12 g/dl	12.4 g/dl	12.8 g/dl
Platelet count	171000/µL	151000/µL	151000/µL	149000/µL	150000/µL

**Figure 1:**
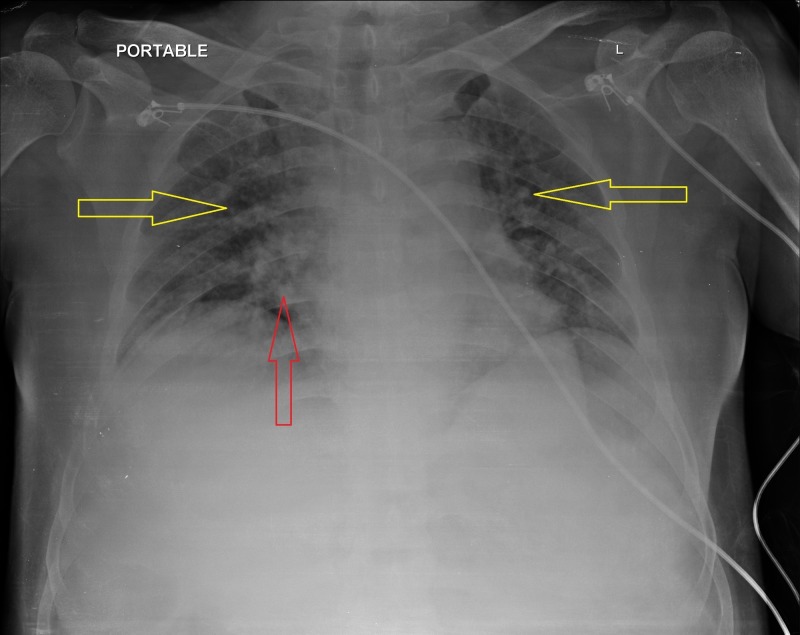
Chest X-ray Red arrow: Hilar and perihilar interstitial prominences. Yellow arrows: Bilateral nodular opacities.

The patient underwent an extensive workup to determine the etiology of his generalized weakness and hypotension. An initial diagnosis of septic shock was ruled out following normal blood and urine cultures. A secondary explanation for the elevated total leukocyte count (TLC) with neutrophilia was attributed to the patient’s previous oral prednisone prescription which was supplemented with hydrocortisone injections in the hospital. Due to the previous history of uveitis, and a mild elevation of angiotensin-converting enzyme (ACE) in the blood, we suspected sarcoidosis as a possible cause of his condition, which was further supplemented by the findings on chest x-ray (CXR), which revealed bilateral hilar and perihilar interstitial prominences with bilateral nodular opacities in the middle and lower lung zones (Figure 1).

We ruled out this possibility owing to the patient’s hasty deterioration and lack of response to the corticosteroid therapy. The patient’s antinuclear antibody (ANA) was negative, and an antineuronal profile ruled out Lambert-Eaton syndrome, myasthenia gravis, and other autoimmune polyneuropathies. Echocardiography revealed an ejection fraction of 60%, which ruled out cardiogenic shock.

**Figure 2:**
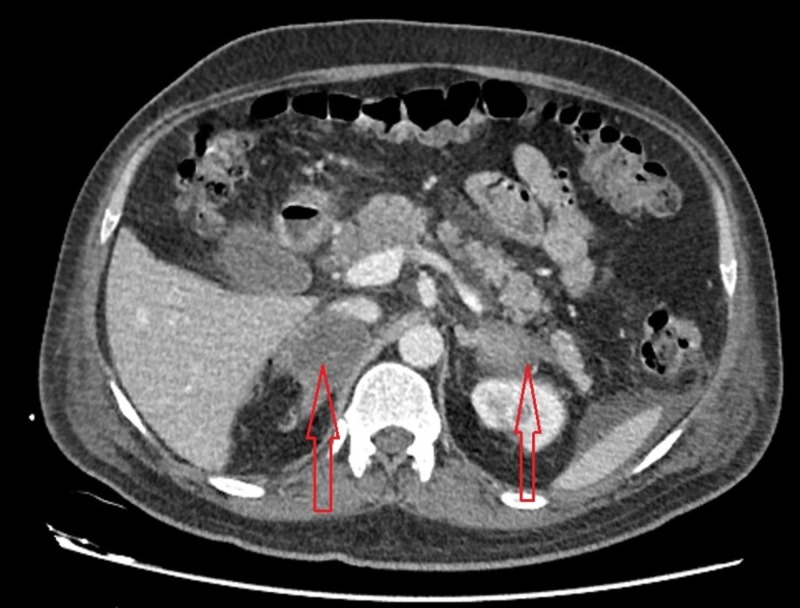
Contrast-enhanced computed tomography (CT) scan of the abdomen Red arrows: Enlarged right adrenal gland with a heterogenous hyperdense area. A similar hetrogenous lesion is present in lateral limb of left adrenal gland.

**Figure 3:**
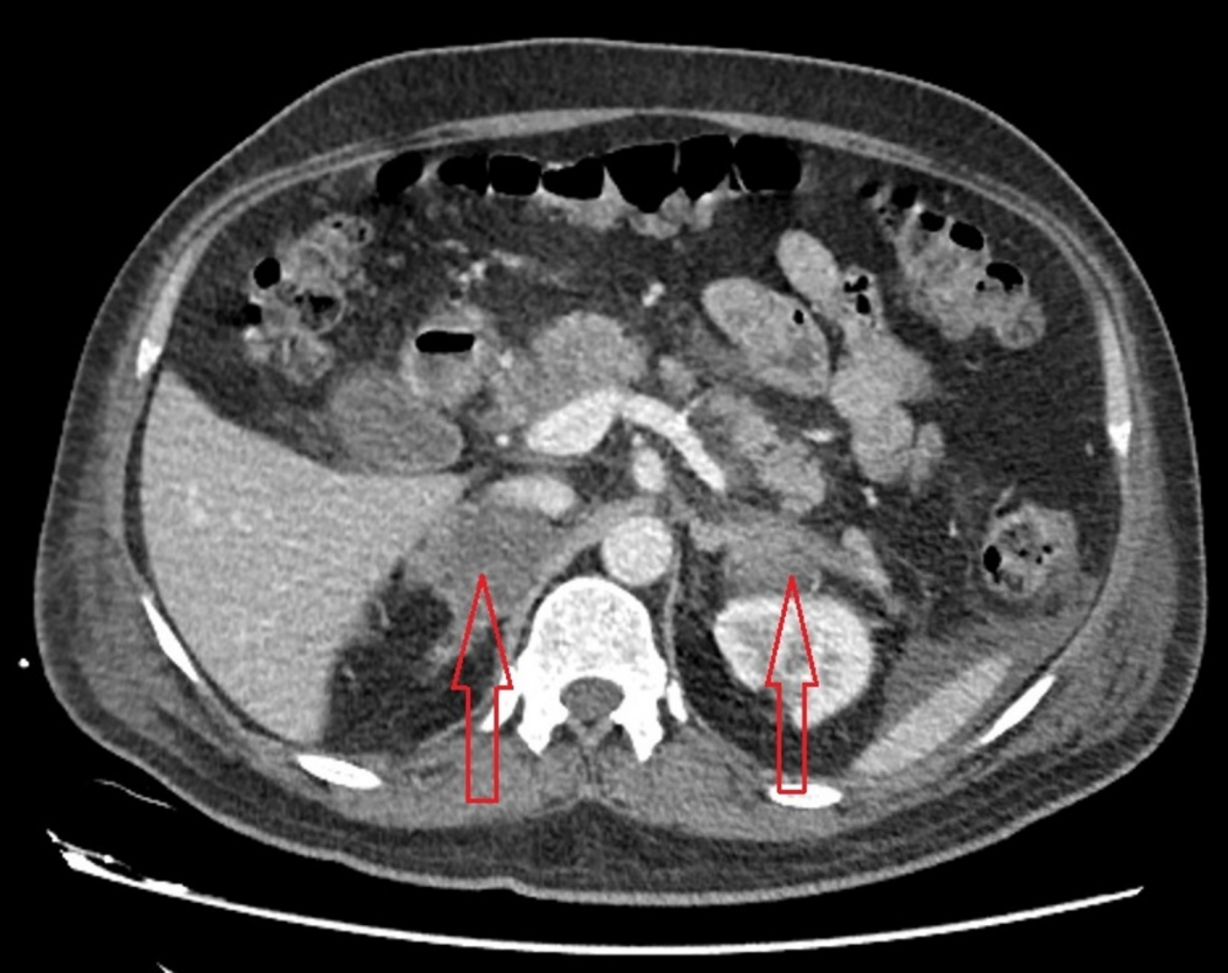
Contrast-enhanced computed tomography (CT) scan of the abdomen Red arrows: Enlarged right adrenal gland with a hetrogenous hyperdense area. A similar hetrogenous lesion is present in lateral limb of left adrenal gland.

A presumptive diagnosis of Addison crisis was made due to the history of steroid use. Further evaluation to support this diagnosis showed an evening serum cortisol of 59 µg/dL, which was still inappropriately low (considering the patient’s clinical predicament). The adrenocorticotropic hormone (ACTH) level was found to be low (4.17 pg/mL) as well. This was followed by a contrast-enhanced computed tomography (CT) scan of the chest, abdomen, and pelvis, which revealed an enlarged right adrenal gland with a heterogeneous hyperdense area. A smaller heterogeneous lesion was present in the lateral limb of the left adrenal gland (Figure 2, Figure 3).

A resultant diagnosis of an adrenal crisis was made owing to the clinical presentation, inappropriately low cortisol level in a hypotensive patient and the presence of bilateral adrenal hemorrhage. An ACTH stimulation test was not performed due to the persistently low blood pressure. An intravenous hydrocortisone drip was initiated at a rate of 200 mg per 24 hours to aggressively manage the adrenal crisis secondary to bilateral adrenal hemorrhage (BAH).

It is important to note that our patient did not have the classical features of adrenal insufficiency. Serum sodium and potassium levels were mildly low, which was consistent with our vigorous fluid resuscitation; low ACTH levels could be justified by the prior prednisone therapy, which could have been further augmented by the administration of hydrocortisone. Another possible differential diagnosis was panhypopituitarism (due to low thyroid stimulating hormone [TSH] and low ACTH levels); however, normal prolactin levels along with the evidence of BAH made the diagnosis less likely. Low TSH, triiodothyronine (T3), and thyroxine (T4) could also be explained by euthyroid sick syndrome, as these levels were obtained when the patient was critically ill.

Further inquiries to determine the causality of BAH ruled out sepsis, owing to a lack of growth in blood and urine cultures. Negative lupus anticoagulant and negative anticardiolipin antibody ruled out antiphospholipid syndrome. Our patient did not have a history of abdominal trauma or recent surgeries. Abdominal CT scan negated the existence of a previous adrenal cyst or signs of previous visceral harm. The patient did not have a history of anticoagulant use, and a coagulation profile ruled out any bleeding diathesis. It was hypothesized that BAH in an otherwise healthy male could have been due to the previous steroid intake. However, no consensus was reached on the etiology of the BAH.

On the third day of admission, the patient was transferred to the intensive care unit (ICU) to seek further therapy for persistently low blood pressure. His condition worsened despite vigorous intravenous resuscitation and vasopressor therapy. He subsequently developed a fever of 101°F with an altered mental status (GCS of 12/15) in the ICU and was therefore intubated. On the fourth day of admission, he developed bradycardia, which ultimately disintegrated into to an asystolic rhythm. A cardiopulmonary resuscitation (CPR) was performed for 25 minutes, which did not revive the patient, and care was ultimately withdrawn.

## Discussion

BAH is a rare ailment that occurs acutely in most cases and carries a relatively high mortality rate of 15% [2]. It is associated with illnesses such as severe sepsis, congestive heart failure, autoimmune etiologies such as antiphospholipid syndrome, the use of anticoagulants; especially in the setting of heparin-induced thrombocytopenia (HIT), as well as severe physical stress such as trauma and surgery. While affiliations between such influences and BAH are well recognized, BAH of an idiopathic nature has also been reported in previous works of literature. Naveen Dhawan et al. presents one such case of an elderly patient, who presented with nonspecific symptoms, making the diagnosis of adrenal insufficiency challenging, much like our case [1-2].

Regardless of the etiology, the exact mechanism of adrenal hemorrhage has not been established. Postulated theories implicate anatomical causes as the usual culprits. An extremely high rate of blood flow, an arterial network that abruptly transitions to a capillary bed, and drainage by a single, central adrenal vein may predispose to adrenal gland hemorrhage [3]. The vessels may become overwhelmed by the voluminous blood flow, which may cause a subsequent schism in its wall, leading to a hemorrhage. A high rate of blood flow may occur secondary to a stressful situation, such as trauma, surgery, severe burns, or sepsis. This leads to stress-induced catecholamine release, which induces vasoconstriction and promotes platelet aggregation, leading to subsequent thrombosis. A clot can cause a hemorrhage due to the pressure effect of the blood accumulating upstream or secondary to tissue damage following a reperfusion injury. Other implicated factors include hypercoagulable states such as antiphospholipid syndrome, spasm of the adrenal vein and age-related hardening of the arterial blood supply [3-4].

The clinical presentation of acute adrenal insufficiency secondary to an adrenal hemorrhage is diverse, as most patients present with non-specific signs and symptoms. Fever is the most common clinical presentation of an adrenal hemorrhage and is found in 70% of the cases [5]. This is inconsistent with our case, where fever developed on the third day of hospitalization, following a period of generalized body weakness for two weeks prior to his arrival, in which the patient remained afebrile. Other features include fatigue, abdominal pain, fever, nausea, and vomiting.

Common laboratory derangements in the setting of acute adrenal insufficiency include hyperkalemia, hyponatremia, hypocalcemia, and hypermagnesemia. While this is true for most patients, textbook laboratory irregularities may not be present in every case [2]. Our patient had a normal serum sodium level through the duration of his admission, while a decreasing trend was appreciated for serum potassium, which is contrary to the classic laboratory manifestations of acute adrenal insufficiency.

As the clinical presentation and laboratory investigations for BAH are dynamic, the role of proper imaging modalities in establishing a diagnosis cannot be overstressed. BAH has differing depictions on an abdominal computed tomography (CT) scan. Most manifestations include a diminished attenuation of the adrenal gland with respect to the adjacent tissues, such as the liver and spleen, along with a thickening of the ipsilateral crura, which may occur secondary to the extension of the blood around the posterior aspect of the kidney. Other common findings range from distinct, well defined, enlarging masses of variant sizes to blatant hemorrhages obliterating adrenal architecture [6].

Magnetic resonance imaging (MRI) of the adrenal glands has a greater accuracy for identifying adrenal hemorrhages and is advantageous in comparison to the traditional CT because it can easily differentiate an adrenal hematoma from the adjacent necrotic tissue, as well as determine the age of the hematoma with respect to its onset. However, CT scan is the investigation of choice to evaluate acute adrenal crisis, especially in hemodynamically unstable patients [1,5].

The deviations in the clinical presentation and laboratory values from the norm led to the delay in diagnosis and a resultant deferral in the prompt initiation of treatment in our patient. Owing to a lack of infectious and hypercoagulable etiology, we concluded that our patient suffered from idiopathic bilateral adrenal hemorrhage (IBAH); even though, we also postulate that the adrenal hemorrhage in our patient may have occurred secondary to corticosteroid use for his uveitis (considering that the consumption of corticosteroids was the only appreciable liability in an otherwise healthy individual).

There are exceptionally rare reports of steroid-induced BAH in literature. Alkhiari et al. described the case of an 87-year-old woman with unilateral adrenal hemorrhage associated with pyelonephritis and lengthy steroid use [7]. Similarly, Lundstrom et al. described a case of BAH in a postoperative patient with a history of prior glucocorticoid use [8]. Similarly, Mudenha et al. described various risk factors for non-traumatic BAH including exogenous steroids [9].

The exact mechanism to explain the correct role of steroids has not been elucidated. However, glucocorticoids have been known to increase blood pressure by manipulating the cardiovascular system in an array of different ways. They are known to accentuate the flow across the glomerular filtration barrier, which leads to glomerular hypertension. They also increase the synthesis of potent vasoconstrictors such as angiotensinogen and atrial natriuretic peptide (ANP), while concomitantly decreasing the synthesis of prostaglandins, which further tightens the vascular diameters, ultimately leading to higher blood pressure. Corticosteroids have also been linked to thromboembolic complications [10]. While a constellation of such events ultimately leading to an adrenal hemorrhage is conceivable, we emphasize the need for an extensive literature review to associate similar cases with this postulation.

The preferred treatment for BAH is intravenous hydrocortisone (100 mg bolus, followed by 200 mg per day via continuous intravenous infusion) and fluid resuscitation with normal saline. Despite treatment, the mortality rate of BAH is as high as 15%, which could be higher if there is a delay in diagnosis and subsequent initiation of the recommended treatment [11-12].

## Conclusions

The clinical depiction and biochemical trends in a case of bilateral adrenal hemorrhage (BAH) can be extremely vague, as illustrated by our findings. Considering the high morbidity and mortality, it is imperative for clinicians to have a high grade of suspicion for this clinical entity. While many etiological factors could precipitate BAH, there are cases where no cause could be elucidated. Our case underscores the importance of considering BAH as an important antecedent to severe hypotension that is not receptive to robust treatment. In such circumstances, a timely diagnosis for BAH using abdominal computed tomography (CT) scan can hasten appropriate therapy and avoid life-threatening complications.
